# The Role of Inbreeding in the Extinction of a European Royal Dynasty

**DOI:** 10.1371/journal.pone.0005174

**Published:** 2009-04-15

**Authors:** Gonzalo Alvarez, Francisco C. Ceballos, Celsa Quinteiro

**Affiliations:** 1 Departamento de Genética, Facultad de Biología, Universidad de Santiago de Compostela, Santiago de Compostela, La Coruña, Spain; 2 Fundación Pública Gallega de Medicina Genómica, Hospital Clínico Universitario, Santiago de Compostela, La Coruña, Spain; Max Planck Institute for Evolutionary Anthropology, Germany

## Abstract

The kings of the Spanish Habsburg dynasty (1516–1700) frequently married close relatives in such a way that uncle-niece, first cousins and other consanguineous unions were prevalent in that dynasty. In the historical literature, it has been suggested that inbreeding was a major cause responsible for the extinction of the dynasty when the king Charles II, physically and mentally disabled, died in 1700 and no children were born from his two marriages, but this hypothesis has not been examined from a genetic perspective. In this article, this hypothesis is checked by computing the inbreeding coefficient (*F*) of the Spanish Habsburg kings from an extended pedigree up to 16 generations in depth and involving more than 3,000 individuals. The inbreeding coefficient of the Spanish Habsburg kings increased strongly along generations from 0.025 for king Philip I, the founder of the dynasty, to 0.254 for Charles II and several members of the dynasty had inbreeding coefficients higher than 0.20. In addition to inbreeding due to unions between close relatives, ancestral inbreeding from multiple remote ancestors makes a substantial contribution to the inbreeding coefficient of most kings. A statistically significant inbreeding depression for survival to 10 years is detected in the progenies of the Spanish Habsburg kings. The results indicate that inbreeding at the level of first cousin (*F* = 0.0625) exerted an adverse effect on survival of 17.8%±12.3. It is speculated that the simultaneous occurrence in Charles II (*F* = 0.254) of two different genetic disorders: combined pituitary hormone deficiency and distal renal tubular acidosis, determined by recessive alleles at two unlinked loci, could explain most of the complex clinical profile of this king, including his impotence/infertility which in last instance led to the extinction of the dynasty.

## Introduction

The Habsburg dynasty (also known as the House of Austria) was one of the sovereign dynasties of Europe. The Spanish branch of this dynasty ruled over the Spanish kingdoms from 1516 to 1700. Under Habsburg rule, Spain reached the zenith of its influence and power in Europe and the world-wide Spanish Empire reached its apogee. The last king of the Spanish Habsburg dynasty was Charles II. He was physically disabled, mentally retarded and disfigured. He proved impotent since no children were born from his two marriages. When Charles II died in 1700 the line of the Spanish Habsburgs died with him and a new dynasty- the French Bourbons- was installed in Spain. In the historical literature it is frequently speculated that strong preference for consanguineous marriages within the Spanish Habsburg line contributed to its extinction. In order to keep their heritage in their own hands, the Spanish Habsburgs began to intermarry more and more frequently among themselves and the result, in a few generations, was a fatal inbreeding that brought the male line of the Spanish Habsburgs to extinction [1]–[5]. This hypothesis of inbreeding, the consequence of the mating of relatives, as a major factor responsible for the extinction of the Spanish dynasty is very suggestive but is based on historical data which have not been examined from a genetic perspective. This is precisely the main goal of the present study.

Three lines of evidence support the inbreeding hypothesis. First of all, the incidence of consanguineous marriages in the Spanish Habsburgs was remarkable (Table 1). The Spanish Habsburg dynasty was founded by Philip the Fair (Philip I), son of the Holy Roman Emperor Maximilian I, by marrying Joanna the Mad (Joanna I of Castile and Aragon) daughter of the Catholic kings, Ferdinand of Aragon and Elizabeth of Castile and during a period of approximately two hundred years a total of 11 marriages were contracted by the Spanish Habsburg kings. Most of these marriages were consanguineous unions: two uncle-niece marriages (Philip II with his niece Anna of Austria and Philip IV with his niece Mariana of Austria), one double first cousin marriage (Philip II with his first wife Mary of Portugal), one first cousin marriage (Charles I, Holy Roman Emperor as Charles V, with Isabella of Portugal), two first cousins once removed marriages, one second cousin marriage and two third cousin marriages. In total, 9 (81.8%) of 11 marriages were consanguineous unions in a degree of third cousins or closer. Second, king Charles II suffered from many different disorders during his life and some of them might be the result of the increase in homozygosity due to inbreeding since the progeny of closely consanguineous couples has an increased incidence of disorders and, in general, detrimental health effects caused by the expression of rare, deleterious recessive alleles inherited from common ancestors [6]–[8]. Third, infant and child mortality was very high in the Spanish Habsburg families. From 1527 to 1661, when Philip II and Charles II were born respectively, the Spanish royal families had 34 children, 10 (29.4%) of them died before 1 year, and 17 (50.0%) of these children died before 10 years [5]. These figures are clearly higher than the mortality rates registered for contemporary Spanish villages which include families belonging to a wide range of social classes and where, for example, infantile mortalities were about 20% [5]. These data suggest that inbreeding depression for infant and child survival could be occurring in the Spanish Habsburg families as a consequence of prolonged consanguineous marriages. It must be emphasized, however, that all these evidences supporting the hypothesis of inbreeding as an important factor in the extinction of the Spanish Habsburg lineage are indirect and, therefore, they are not conclusive. Thus, it has not been demonstrated that the physical and mental disabilities suffered by the king Charles II were caused by the expression of detrimental recessive alleles inherited from common ancestors. These detrimental health effects could be due to either environmental causes or genetic effects which are not associated with consanguinity and the same argument is applicable for the high mortalities experienced by the Spanish Habsburg families. With respect to the high incidence of consanguineous marriages in the Spanish Habsburg dynasty it is necessary to take into account that, from a Western perspective, marriages between close biological relatives is generally regarded with suspicion and distaste, reflecting historical and religious prejudice. However, contrary to widespread opinion in Western countries, consanguinity is widely preferential in present large human populations of Asia and Africa where consanguineous marriages currently account for approximately 20–50% of all unions (in the computation of these percentages those unions contracted between individuals biologically related as second cousins or closer are categorized as consanguineous) [9]–[11]. The highest levels of inbreeding in major populations have been found in urban Pondicherry (South India) and among army families in Pakistan where 54.9% and 77.1% of marriages are consanguineous, respectively [12]–[13]. In Pondicherry 20.2% of marriages are uncle-niece and 31.3% first cousins, whereas in the Pakistan study 62.5% of marriages are between first cousins. Therefore, in the light of these data, the incidence of consanguineous marriages in the Spanish Habsburg dynasty does not seem so extreme when is considered in a general context of inbreeding in human populations.

**Table 1:** The Spanish Habsburg kings and their marriages.

KING	QUEEN	Type of consanguineous marriage
Philip I (1478–1506)	Joanna I of Castile and Aragon	Third cousins
Charles I (1500–1558)	Isabella of Portugal	First cousins
Philip II (1527–1598)	Mary of Portugal	Double first cousins
	Mary I of England	First cousins once removed
	Elizabeth of Valois	Remote kinship
	Anna of Austria	Uncle-niece
Philip III (1578–1621)	Margaret of Austria	First cousins once removed
Philip IV (1605–1665)	Elizabeth of Bourbon	Third cousins
	Mariana of Austria	Uncle-niece
Charles II (1661–1700)	Marie Louise d'Orleans	Second cousins
	Maria Anna of Neuburg	Remote kinship

## Methods

**Figure 1:**
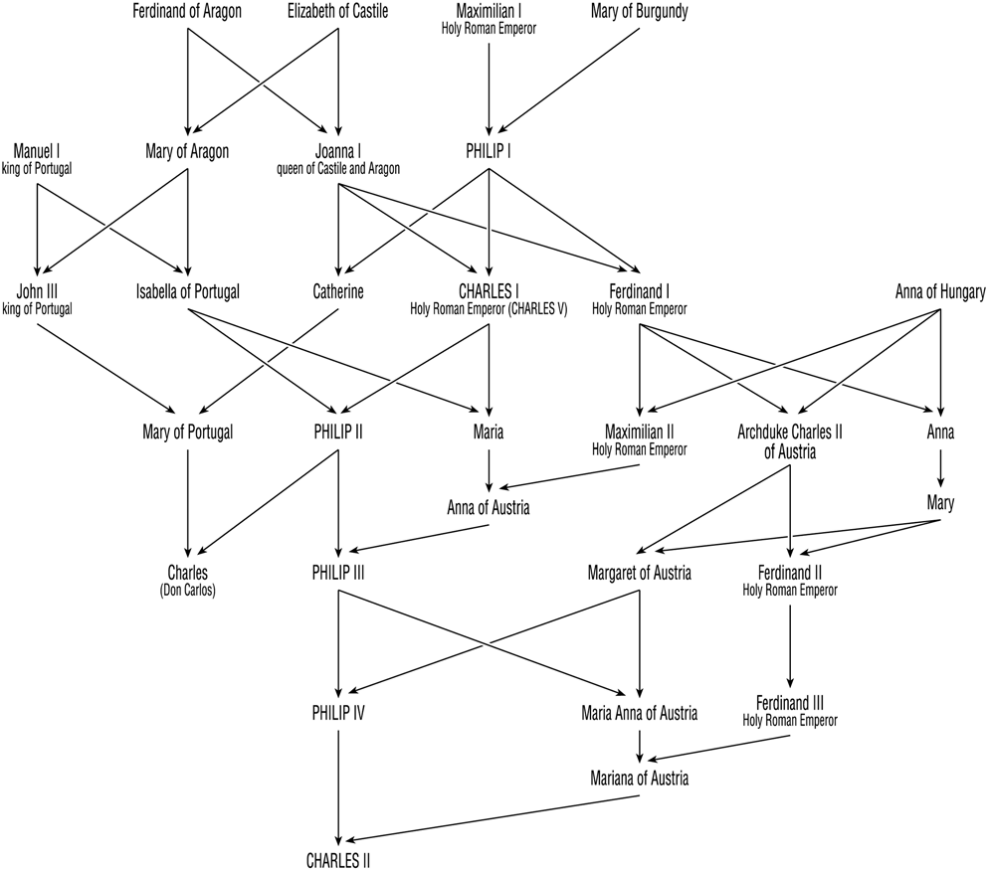
Pedigree of the Spanish Habsburg kings (in capital letters).

Genealogical information of the Spanish Habsburg kings obtained from several sources [1]–[3], [14]–[15] was introduced into a data base which included more than 3,000 individuals along 16 parent-offspring generations (Fig. 1 shows a partial pedigree of the Spanish Habsburg kings represented by chains of descent). The data base was used for computing the inbreeding coefficient *F* (probability that an individual receives at a given locus two genes identical by descent due to the common ancestry between his parents) of the Spanish Habsburgs by means of the FSpeed computer programme [16].

In order to evaluate the impact of inbreeding on survival in the Spanish Habsburg dynasty, mortality data of progenies of eight royal families were obtained from several sources [17]–[18]: the families of the Spanish Habsburg kings Philip I, Charles I, Philip III, two families of king Philip II with his third and fourth wives (Elizabeth of Valois and Anna of Austria) and the two families of king Philip IV. In addition, the family of Ferdinand of Aragon and Elizabeth of Castile, parents of Joanna I of Castile and Aragon, is also considered in order to increase the number of studied families. The families of the king Philip II with his first and second wives (Mary of Portugal and Mary I of England) are not considered since Mary of Portugal had only a single child (Charles) and there was no child in the marriage between Philip II and Mary I of England (Mary Tudor). Mortality data of progenies of the eight royal families were classified into three categories: late miscarriages and stillbirths, neonatal deaths (deaths in the first month of life) and deaths between month 1 and year 10. As a whole, there were 51 pregnancies in the eight families: 5 miscarriages and stillbirths, 6 neonatal deaths, 14 deaths between month 1 and year 10 and 26 survivals at age 10, and the mean number of pregnancies per family was 6.38±0.42. Multiple regression analyses and other statistical methods were performed by means of the SPSS 15.0 statistical software system.

## Results and Discussion

**Figure 2:**
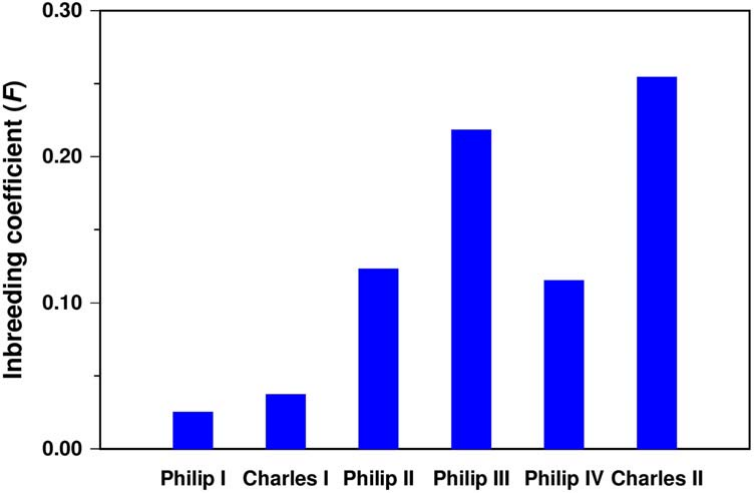
Inbreeding coefficient (*F*) of the Spanish Habsburg kings.

**Figure 3:**
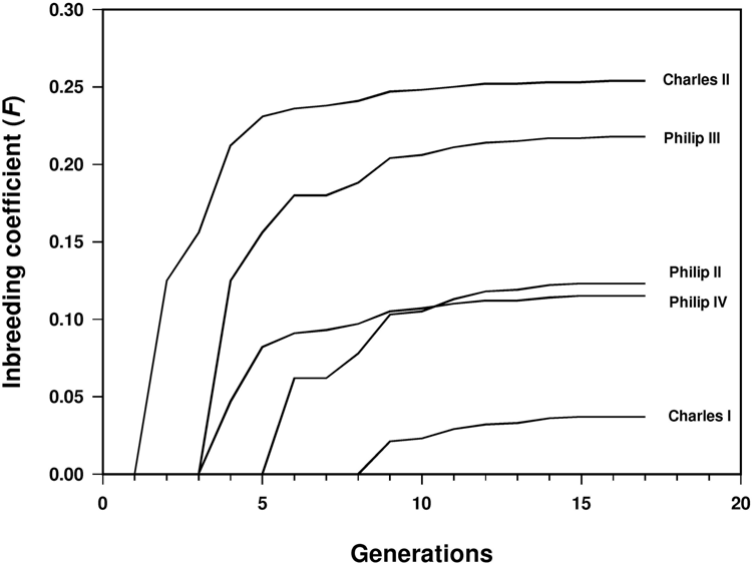
Increase in inbreeding coefficients (*F*) with increasing depth of pedigree in Spanish Habsburg kings.

In order to assess the possible effects of inbreeding on the Spanish Habsburg dynasty the inbreeding coefficient (*F*) of the Spanish Habsburg kings must be calculated. Inbreeding coefficients of the Spanish Habsburgs were computed from an extended pedigree traced back as far as 16 ancestral generations from the last Spanish Habsburg king and involving more than 3,000 individuals (a partial pedigree including only a few generations is represented by chains of descent in Fig. 1). Computation of inbreeding coefficients on extended pedigrees is critical in the present study since remote or ancestral consanguinity can make a substantial contribution to the inbreeding of a given individual and the exploration of pedigrees limited to a shallow depth carries the risk of considerably underestimating the degree to which individuals are inbred [19]–[20]. In addition to the Spanish Habsburgs, our extended pedigree includes a number of individuals from many other different European royal houses such as the Austrian Habsburgs, the Avis dynasty of Portugal and the Valois and Bourbon dynasties of France since most of the European dynasties of that time were closely interconnected as a consequence of political alliances. As shown in Table 2, the inbreeding coefficient of the Spanish Habsburg kings increased strongly along generations from 0.025 (Philip I) to 0.254 (Charles II) and the average *F* value is 0.129 (Figure 2). The inbreeding coefficients of the kings' wives are clearly lower since they vary between 0.001 (Elizabeth of Valois) and 0.155 (Mariana of Austria) with an average of 0.070. The highest values of the inbreeding coefficient in the Spanish Habsburgs correspond to kings Charles II (0.254) and Philip III (0.218), and the crown prince Charles (Don Carlos, 0.211), son of Philip II and his first wife Mary of Portugal. It is not surprising that these three individuals present the highest values of inbreeding coefficient among the Spanish Habsburgs since they are sons of either uncle-niece (Charles II and Philip III) or double first cousin (prince Charles) marriages. However, the inbreeding coefficient for these three individuals is practically twice the expected value for those types of consanguineous marriages (*F* = 0.125 in both cases) and very close to the expected value in an incestuous union as parent-child or brother-sister (*F* = 0.250 in both cases). The development of the inbreeding coefficients with increasing pedigree depth shows that the inbreeding levels for most Spanish Habsburg kings are not definitely established until pedigrees of at least 10 generations depth are analyzed (Fig. 3). This means that ancestral consanguinity from multiple remote ancestors makes a substantial contribution to the inbreeding coefficient of the Spanish Habsburg kings and the contribution of this remote consanguinity is very similar in magnitude to that due to close consanguinity (uncle-niece or first cousin marriages). Therefore, the inbreeding of the Spanish Habsburg kings was not only the consequence of a few generations of unions between close relatives as it is sometimes claimed [1]. It is also interesting to note that some particular Spanish Habsburg families had relatively small levels of inbreeding. In particular, the progenies of Philip II and his third wife Elizabeth of Valois (*F* = 0.008), and Philip IV and his first wife Elizabeth of Bourbon (*F* = 0.050). Both marriages did not give place to a king since, in the first case, only daughters were born and, in the second, the heir to the throne, the prince Baltasar Charles, died at early age. These historical contingencies were relevant for the Spanish Habsburg dynasty since when queens Elizabeth of Valois and Elizabeth of Bourbon died, Philip II and Philip IV married their nieces, Anna and Mariana, respectively, and consequently an important increase in the inbreeding coefficient of the resulting progenies was produced.

**Table 2:** Inbreeding coefficient (*F*) of the Spanish Habsburg kings, their wives and their progenies.

	KING	KING'S WIFE	OFFSPRING
Philip I	0.025	0.039	0.037
Charles I	0.037	0.101	0.123
Philip II	0.123	0.123	0.211
		0.008	None
		0.001	0.008
		0.106	0.218
Philip III	0.218	0.139	0.115
Philip IV	0.115	0.007	0.050
		0.155	0.254
Charles II	0.254	0.078	None
		0.008	None
Mean	0.129	0.070	0.127
SD	0.093	0.059	0.092

**Figure 4:**
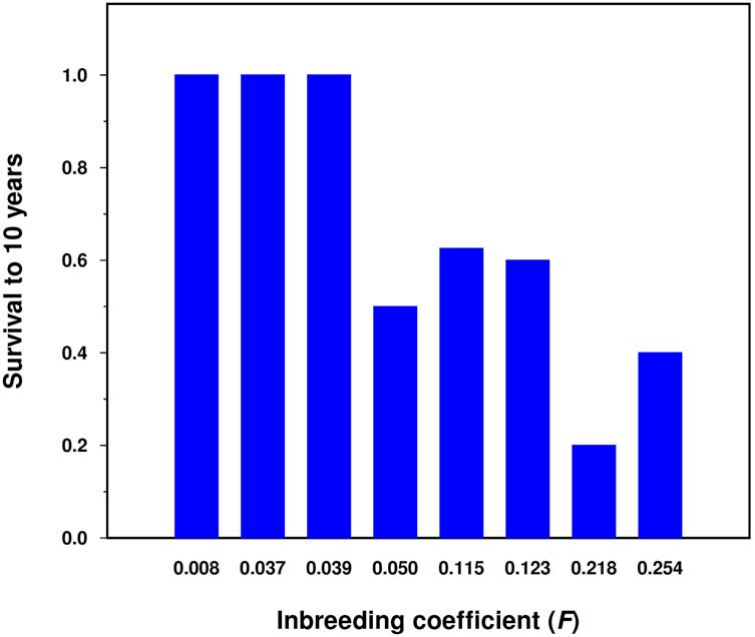
Survival to 10 years (prenatal and neonatal deaths not included) and inbreeding coefficient (*F*) in eight progenies of Spanish kings.

The effect of inbreeding on survival in the Spanish Habsburg dynasty is investigated from mortality data of eight royal families (Table 3). Two different regression analyses of survival to 10 years of the progenies of the eight royal families as a function of the inbreeding coefficients of the offspring and mother have been performed. One of these analyses is a multiple regression analysis computed by ordinary least squares and the other is a multiple logistic regression analysis based on logit-transformed data where parameter estimation is performed by maximum likelihood methods. When all pre- and postnatal losses are included in the calculation of survival to 10 years, a statistically significant effect of the inbreeding coefficient of the offspring on survival is detected by the multiple logistic regression and this inbreeding effect is very close to the statistical significance (P = 0.053) by the multiple regression by ordinary least squares (Table 4). When miscarriages, stillbirths and neonatal deaths are removed from the analysis, the inbreeding effect on survival to 10 years is more consistent and both multiple and logistic regressions show a clear inbreeding depression in the inbred offspring (Table 4 and Figure 4). On the other hand, an effect of maternal inbreeding on survival to 10 years is not detected by the regression analyses. These results suggest that the main effect of inbreeding on survival is produced in children between month 1 and year 10. In fact, it seems that inbreeding does not have a significant effect on early mortality (prenatal and neonatal deaths) in the Spanish Habsburg dynasty. Thus, a negative statistical association between prenatal losses (miscarriages and stillbirths) and inbreeding coefficient among families is detected by Kendall's coefficient of rank correlation but this association is not statistically significant (τ = −0.161, P = 0.595). Similarly, the association between neonatal deaths and inbreeding coefficient among families is not statistically significant (τ = −0.041, P = 0.833). These slight and non significant negative associations between early mortalities and inbreeding coefficient may be easily explained as a consequence of sampling errors due to small sample size (51 pregnancies in the eight families studied) and the possibility that inbreeding plays a protective role in early mortality must be considered a very unlikely hypothesis. The magnitude of the inbreeding depression in the Spanish Habsburg dynasty seems to be remarkable. Thus, under a simple linear regression model, the regression coefficient of survival to 10 years (prenatal and neonatal deaths not included) as a function of the inbreeding coefficient is −2.850±0.756 which is significantly lower than 1 (t = 3.771, P = 0.009 by a two sided test). This supposes that the impact of an *F* = 0.0625, corresponding to the progeny from a first cousin marriage, on survival is 17.8%±12.3. In present human populations the most recent estimate of inbreeding effect on the children of first cousin marriages is 4.4%±4.6 (depression of survival at age 10 including late miscarriages, stillbirths and neonatal deaths) based on a large data set of first cousins [21]. Although these two figures are not directly comparable since one refers to an inbreeding effect in a particular family and the other refers to an average effect for a large number of families in different populations they show that the reduction in survival at age 10 for a first cousin progeny with respect to the offspring from unrelated parents detected in the Spanish Habsburg families is very remarkable.

**Table 3:** Inbreeding coefficient (*F*), mortality and survival of eight progenies of Spanish kings.

King/Queen	*F*	Total number of pregnancies	Miscarriages and stillbirths	Neonatal deaths	Deaths between month 1 and year 10	Survivals at age 10	Survival to 10 years (all pre- and postnatal losses included)	Survival to 10 years (miscarriages, stillbirths and neonatal deaths not included)
Ferdinand of Aragon								
Elizabeth of Castile	0.039	7	2	0	0	5	0.714	1.000
Philip I								
Joanna I	0.037	6	0	0	0	6	1.000	1.000
Charles I								
Isabella of Portugal	0.123	7	1	1	2	3	0.429	0.600
Philip II								
Elizabeth of Valois	0.008	4	1	1	0	2	0.500	1.000
Anna of Austria	0.218	6	1	0	4	1	0.167	0.200
Philip III								
Margaret of Austria	0.115	8	0	0	3	5	0.625	0.625
Philip IV								
Elizabeth of Bourbon	0.050	7	0	3	2	2	0.286	0.500
Mariana of Austria	0.254	6	0	1	3	2	0.333	0.400

**Table 4:** Multiple regression analyses for survival to 10 years as a function of the inbreeding coefficient (*F*) of progeny and mother in eight families of Spanish kings.

	Multiple regression by least squares	P	Multiple logistic regression	P
Survival to 10 years (all pre- and postnatal losses included):				
Constant	0.625	0.004	0.557	0.282
*F* offspring	−4.158	0.053	−18.079	0.013
*F* mother	4.315	0.146	17.894	0.080
Survival to 10 years (miscarriages, stillbirths and neonatal deaths not included):				
Constant	0.930	0.000	2.128	0.009
*F* offspring	−4.174	0.034	−17.004	0.027
*F* mother	2.363	0.331	6.134	0.604

As already mentioned, Charles II, the last king of the Spanish Habsburg dynasty, presented important physical and mental disabilities suffering from a number of different diseases during his life [4]–[5], [17], hence being known in Spanish history as *El Hechizado* (“The Hexed”). According to contemporary writings, he was often described as “big headed” and “weak breast-fed baby”. He was unable to speak until the age of 4, and could not walk until the age of 8. He was short, weak and quite lean and thin. He was described as a person showing very little interest on his surroundings (abulic personality). He first marries at 18 and later at 29, leaving no descendency. His first wife talks of his premature ejaculation, while his second spouse complaints about his impotency. He suffers from sporadic hematuria and intestinal problems (frequent diarrhea and vomits). He looked like an old person when he was only 30 years old, suffering from edemas on his feet, legs, abdomen and face. During the last years of his life he barely can stand up, and suffers from hallucinations and convulsive episodes. His health worsens until his premature death when he was 39, after an episode of fever, abdominal pain, hard breathing and comma. In the light of the knowledge of the current clinical genetics and on information gathered by the historians on the health of Charles II we might speculate on the origin of his illness. Although we recognize that it is highly speculative, some of the health problems suffered by Charles II could have been caused by the action of detrimental recessive genes given his high inbreeding coefficient (*F* = 0.254) with 25.4% of his autosomal genome expected to be homozygous. In this sense, the simultaneous occurrence in Charles II of two genetic disorders determined by recessive alleles, combined pituitary hormone deficiency (CPHD, OMIM 26260) and distal renal tubular acidosis (dRTA, OMIM 602722), could explain an important part of his complex clinical profile. The most frequent genetic cause of hereditary CPHD is mutations in *PROP1* (5q) that cause a multiple endocrine deficit of pituitary hormones [22]–[24]. This anomalous hormonal phenotype would lead to development of short stature, hypotonia and muscular weakness and abulic personality as well as infertility/impotence and gastrointestinal symptoms which characterize much of the clinical profile of Charles II. Any additional physical stress will exacerbate these clinical manifestations, often resulting in intense abdominal pain, fever and lethargy followed by hypovolemic vascular collapse [25]–[26]. dRTA is a rare renal disease associated with alterations of the urine acidification mechanisms and may be caused by recessive mutations in *ATP6V0A4* (7q) or *ATP6V1B1* (2q) genes [27]–[28]. In Charles II, his muscular weakness at a young age, rickets, hematuria and his big head relative to his body size could be attributed to this genetic disorder. In this way, we may speculate that most of the symptomatology showed by Charles II could be explained by two different genetic disorders produced by detrimental recessive alleles at two unlinked loci. Evidently, the probability of an affected individual suffering from two very rare recessive traits must be very low but it must be taken into account that inbreeding may cause the association of two recessive traits even for unlinked loci [29]. In addition, recent studies with SNPs and microsatellite markers have shown that the amount of genomic homozygosity of consanguineous individuals is often greater than expected from pedigree information [30], suggesting that the genomic homozygosity of Charles II could have been higher than the inferred 25.4%.
